# Phenylpropanoids in *Silybum marianum* cultures treated with cyclodextrins coated with magnetic nanoparticles

**DOI:** 10.1007/s00253-022-11886-2

**Published:** 2022-03-28

**Authors:** Purificación Corchete, Lorena Almagro, Jose Antonio Gabaldón, María Angeles Pedreño, Javier Palazón

**Affiliations:** 1 Departamento de Botánica y Fisiología Vegetal, Facultad de Biología, Universidad de Salamanca, Salamanca, Spain; 2 Departamento de Fisiología Vegetal, Facultad de Biología, Universidad de Murcia, Murcia, Spain; 3 Departamento de Tecnología de la Alimentación y Nutrición, Universidad Católica San Antonio de Murcia, Murcia, Spain; 4 Laboratori de Fisiologia Vegetal, Facultat de Farmacia, Universitat de Barcelona, Barcelona, Spain

**Keywords:** *Silybum marianum* suspension cultures, Hydroxypropyl-β-cyclodextrin polymer-coated magnetic nanoparticles, Methyl jasmonate, *t*-resveratrol, Naringenin

## Abstract

**Abstract:**

The glucose oligosaccharide-derived cyclodextrins (CDs) are used for improving bioactive compound production in plant cell cultures because, in addition to their elicitation activity, CDs promote product removal from cells. However, despite these advantages, the industrial application of CDs is hampered by their high market price. A strategy to overcome this constraint was recently tested, in which reusable CD polymers coated with magnetic Fe_3_O_4_ nanoparticles were harnessed in *Vitis vinifera* cell cultures to produce *t*-resveratrol (*t*-R).

In this study, we applied hydroxypropyl-β-CDs (HPCD) and HPCDs coated with magnetic nanoparticles (HPCD-EPI-MN) in methyl jasmonate (MJ)-treated transgenic *Silybum marianum* cultures ectopically expressing either a stilbene synthase gene (STS) or a chalcone synthase gene (CHS), and compared their effects on the yields of *t*-R and naringenin (Ng), respectively. HPCD-EPI-MN at 15 g/L stimulated the accumulation of metabolites in the culture medium of the corresponding transgenic cell lines, with up to 4 mg/L of *t*-R and 3 mg/L of Ng released after 3 days. Similar amounts were produced in cultures treated with HPCD. Concentrations higher than 15 g/L of HPCD-EPI-MN and prolonged incubation periods negatively affected cell growth and viability in both transgenic cell lines. Reutilization of HPCD-EPI-MN was possible in three elicitation cycles (72 h each), after which the polymer retained 25–30% of its initial efficiency, indicating good stability and reusability.

Due to their capacity to adsorb metabolites and their recyclability, the application of magnetic CD polymers may reduce the costs of establishing efficient secondary metabolite production systems on a commercial scale.

**Key points:**

*• Long-term transgenic S. marianum suspensions stably produce transgene products*

*• t-R and Ng accumulated extracellularly in cultures elicited with HPCD and HPCD-EPI-MN*

*• The recyclability of HPCD-EPI-MN for metabolite production was proven*

**Graphical abstract:**

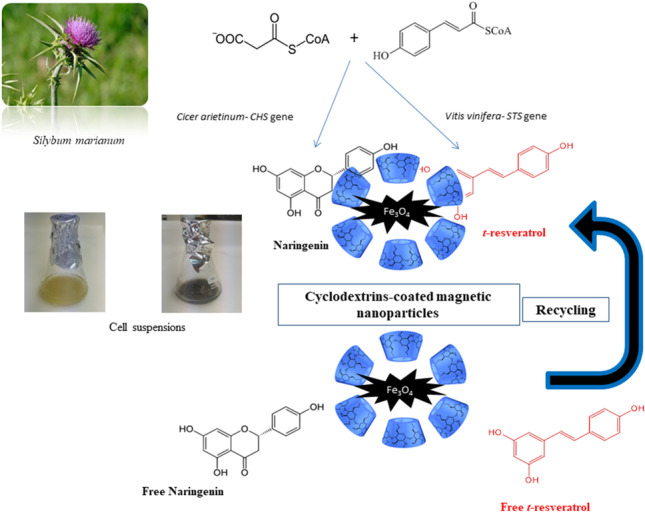

**Supplementary Information:**

The online version contains supplementary material available at 10.1007/s00253-022-11886-2.

## Introduction

Plant cell suspension cultures have long been considered efficient platforms for the production of valuable plant secondary metabolites and an alternative to conventional plant breeding. However, the lack of differentiated cell/tissue structures and a tendency to genetic instability over prolonged culture periods, among other factors, undermine the efficiency of cell suspensions in comparison with the intact plant.

Strategies aimed at optimizing biomass accumulation and biosynthesis include, particularly, elicitation and the application of metabolic engineering approaches (Verpoorte et al. 2002; Lu et al. 2002; Zhao et al. 2005; Narayana and Srivastava 2008; Smetanska 2008; Diamond and Desgagné-Penix 2016; Appelhagen et al. 2018). Another focus of interest for the biotechnological production of secondary metabolites is the extraction of target compounds from the culture medium. Therefore, permeabilizing cells for extracellular accumulation has proved to be a convenient approach to reduce an eventual feedback inhibition of biosynthesis and to facilitate product recovery.

In the last decade, researchers working in the field of secondary metabolite production in cell suspensions have found CDs to be an invaluable tool for increasing productivity. The physico-chemical properties of these cyclic glucose oligosaccharides permit the stabilization and solubilization of complexed molecules; due to their oligosaccharide nature, CDs have elicitor properties; and, depending on the type and concentration, CDs are able to extract intracellular compounds (Bru et al. 2006; Zamboni et al. 2006; Loftsson and Masson 2011; Baeck et al. 2013; Zhang et al. 2013; Landy et al. 2012; Almagro et al. 2016; Jansook et al. 2018; Cardillo et al. 2021).

Pioneering studies on the elicitor effects of CDs were performed in *Vitis vinifera* plant cell suspensions to produce the biologically active stilbene *t*-R (Bru et al. 2006). Subsequently, a number of studies have harnessed the properties of CDs to produce an array of secondary metabolites in plant cell cultures, including phenylpropanoid compounds (Belchi-Navarro et al. 2011; Marsh et al. 2014; Soto-Argel et al. 2018; García-Pérez et al. 2019), terpenes, such as taxol (Cusido et al. 2014) and artemisin (Durante et al. 2011), and terpenoid indole alkaloids (Almagro et al. 2011; Zhou et al. 2015).

Nevertheless, the implementation of these successful results in industrial-scale processes is hindered by the high cost of CDs. A potential solution was recently designed by Almagro et al. (2020), based on recyclable CD polymers. Hydroxypropylated CDs (HPCDs) were crosslinked with epichlorohydrin, and the resulting polymer was coated with magnetic Fe_3_O_4_ nanoparticles (Almagro et al. 2020). When applied to *V. vinifera* suspension cultures in combination with MJ, the CD/Fe_3_O_4_ nanoparticles had a positive effect on *t-*R production, although to a lesser extent than other elicitation strategies. Importantly, however, these magnetic polymers could be reused in at least three elicitation cycles. Despite these promising results, no other application of CD/Fe_3_O_4_ nanoparticles has been reported in the literature to date. Therefore, with the aim of testing the effect of these novel CDs in a different plant species, in the current work, they were applied in metabolically engineered cell cultures of the Asteraceae plant, *Silybum marianum*.

*S. marianum* cell cultures intracellularly store chlorogenic acid derivatives, products of the phenylpropanoid pathway (Sánchez-Sampedro et al. 2007). In a previous study with *S. marianum*, we demonstrated the possibility of increasing metabolic flux toward competing routes by expressing structural enzymes specific to the target pathway. In this manner, stable transgenic cell lines capable of producing *t-*R were generated by introducing a STS from *V. vinifera* in the *S. marianum* genome (Hidalgo et al. 2017). In another recent study, the heterologous expression in *S. marianum* of a CHS gene from *Cicer arietinum* enhanced the production of the bioactive flavanone Ng (Villar et al. 2020). In these two transgenic *S. marianum* cultures, elicitation with MJ was necessary to detect the targeted metabolites, *t*-R and Ng. In addition, application of dimethylated CDs (DMCD) was reported to induce the extracellular accumulation of free *t*-R (Hidalgo et al. 2017), as also occurred in *V. vinifera* cultures (Almagro et al. 2015).

The present work shows the effect of free HPCDs and HPCDs coated with magnetic nanoparticles on *t*-R and Ng production in *STS*- and *CHS*-transformed *S. marianum* cells, respectively, as well as their repercussion on growth and cell viability of the transgenic cultures.

## Material and methods

### Chemicals

MJ and plant culture media were purchased from Sigma, and HPCDs (substitution degree: 4.5) from Cyclolab (Budapest, Hungary). HPCD-EPI polymers coated with Fe_3_O_4_ nanoparticles were synthesized as described previously (Almagro et al. 2020).

### Cell suspensions

Two independently established transgenic suspension cultures of *S. marianum* were used for the experiments. Transgenic suspensions heterologously expressing the coding region of *STS*-3 from *V. vinifera* (Ref. Seq. XM_002263686.2. PREDICTED: stilbene synthase 3 [*Vitis vinifera*]) (*STS*-transformed cultures) were obtained as described in Hidalgo et al. (2017). *CHS*-transformed cultures were generated by genetic transformation with a *CaCHS* clone from *C. arietinum* containing the *CHS* coding region (GenBank: AJ012690.1) (Villar et al. 2020).

For routine subcultures every two weeks, suspensions were maintained in MS medium containing 3% sucrose, 1 mg/L 2,4-dichlorophenoxyacetic acid and 0.5 mg/L 6-benzylaminopurine, as described previously (Sanchez Sampedro et al. 2005). Cultures were incubated in the dark at 25 °C and shaken at 90 rpm. Changes in the cell weight of cultures were periodically checked to monitor cell growth. Cell viability was estimated according to Jones and Senft (1985).

## Elicitation and metabolite analyses

For the elicitation experiments, 1 ± 0.14 g fresh weight 14-day-old cells were transferred to 100 mL flasks containing 20 mL medium of same composition as for maintenance, to which different concentrations (2.5, 7.5, and 15 g/L) of free HPCDs or HPCD-EPI-MN were added together with 100 µM MJ. Metabolite production in cultures was periodically analyzed up to 6 days of treatment.

To recover HPCD-EPI-MN polymers, a magnet was placed on the bottom surface of the culture flasks to separate the HPCD-EPI-MN from the cell suspension, which were filtered through a borosilicate glass funnel. The cells retained in the filters were used for monitoring weight and viability. Metabolites were extracted from filtrates three times with two volumes of ethyl acetate. For metabolite extraction from the magnetic polymers, these were rinsed first with distilled water three times and then extracted with ethyl acetate (1:10 *w/v*) by stirring with a magnetic stirrer for 30 min, the extraction process being repeated three times. The ethyl acetate extracts were collected and evaporated at 40 °C in vacuum. Residues were resuspended in 1 mL methanol and analyzed by HPLC. The medium of cultures not treated with magnetic polymers was separated by filtration and also extracted three times with two volumes of ethylacetate.

The reusability of the HPCD-EPI-MN polymer was evaluated up to three times. For this, cells of the respective transgenic line were inoculated in 20 mL culture medium to which 15 g/l of a previously employed HPCD-EPI-MN polymer was added (second use). This procedure was repeated one more time with the HPCD-EPI-MN polymer employed in the second use (third use). MJ (100 µM) was also aseptically added to flasks in both experiments. Metabolite extraction from magnetic polymers was carried out as described in the previous paragraph.

HPLC analysis was performed in a Spherisorb ODS-2 (5 μm) reversed-phase column (4.6 × 250 mm) at 35 °C. The mobile phase was a mixture of methanol and acetic acid:water (5:55 v/v) with a gradient run initiated with 30:70; a linear increase to 70:30 over 25 min; 5 min 70:30; return to initial conditions in 5 min and stabilization 70:30 for 5 min. Flow rate was 1 ml/min. Chromatograms were acquired at 306 nm (*t*-R) and 288 nm (Ng). Prior to injection, samples were dissolved in the elution solvent. Concentrations of *t*-R and Ng were estimated using the standard curve generated by pure compounds.

## Absorption–desorption of Ng from magnetic polymers

Experiments on the absorption–desorption of *t*-R were carried out in a previous study (Almagro et al. 2020). In the current work, a preliminary test was performed to investigate the capacity of HPCD-EPI-MN to adsorb Ng. Thus, Ng (150 µg from a concentrated stock dissolved in methanol) was loaded into the HPCD-EPI-MN (150 mg) dispersed in 20 mL distilled water. After stirring at 100 rpm for 30 min at 25 °C, HPCD-EPI-MN was separated from the solution by magnetic decantation and extracted with 10 mL of ethyl acetate under continuous stirring with a magnetic stirrer. Samples were analyzed periodically until all the Ng was extracted in the ethereal phase. Extracted Ng levels were monitored by HPLC as described above. The experiments were done in triplicate.

## Statistics

Experiments were repeated twice, each time in triplicate. Standard deviation (± SD) of the means was used to assess the confidence of experimental data. All data obtained was analyzed with statistic software GraphPad Prism Version 5.03. Differences between treatments were evaluated with a one-way variance analysis (ANOVA) with a significance level of 0.05. Multiple (pair-wise) comparisons were done by Tukey’s honest significantly test (Tukey’s HSD) and results were presented as confidence intervals at 95% (95% CI).

## Results

The capacity of HPCDs coated with Fe_3_O_4_ nanoparticles to extract secondary metabolites was investigated in MJ-elicited transgenic *S. marianum* cells. Transgenic *S. marianum* suspension cultures had been established in 2016 (expressing an *STS* gene) and 2018 (heterologously expressing a *CHS* gene from *C. arietinum)*. Both transformed lines have been maintained ever since with no apparent loss of transgene expression, as demonstrated by their capacity to produce the transgene products under appropriate elicitation conditions. This observation is of interest, as stable chromosomal integration and persistent gene expression in vivo is required if plant cell cultures are intended to be commercially exploited.

Cell growth and *t*-R production in *STS*-transformed *S. marianum* cultures treated with HPCD or HPCD-EPI-MN.

In a previous study by Hidalgo et al. (2017), free *t-*R produced in MJ-elicited *STS*-transformed *S. marianum* cultures was detected in the extracellular medium at levels of up to 12 mg/L at 72 h when the cultures were also treated with 30 mM of DMCDs (39.3 g/L culture). In the present work, HPCDs were used instead, given their beneficial effects on *t*-R production in *V. vinifera* cultures. In a preliminary analysis, extracellular *t*-R accumulation was determined in cultures treated with 30 mM of HPCD. An average of 10.63 ± 1.8 mg/L of *t*-R was measured after 3 days (Table S1), thus showing the suitability of this class of modified CD for the experiments. In the first trial with HPCD-EPI-MN, 30 g/L was found to be toxic for the cultures, even at 24 h of treatment; subsequently, the tested concentrations were no higher than 15 g/L.

**Fig. 1:**
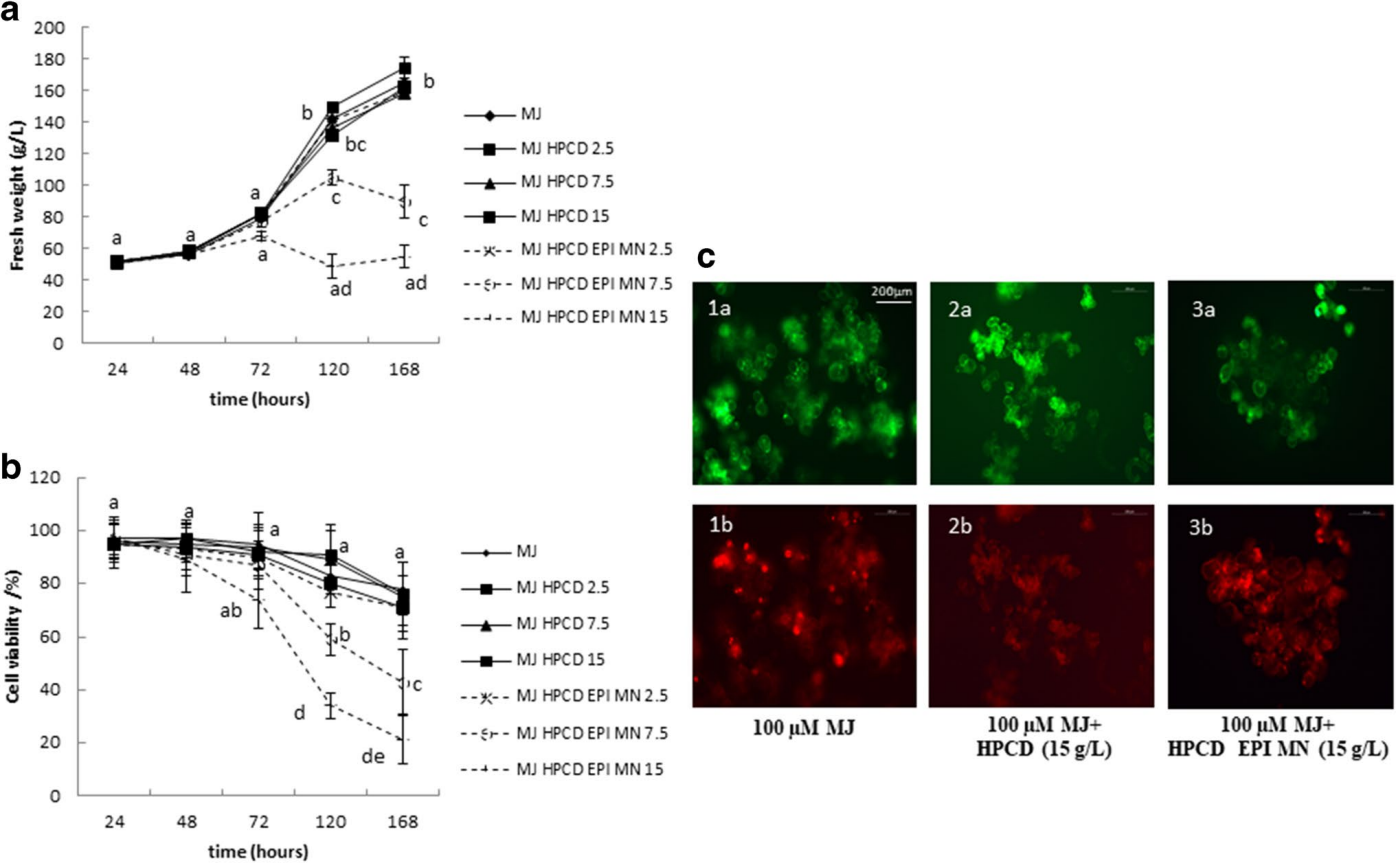
Effect of different concentrations (2.5, 7.5, and 15 g/L) of hydroxypropyl-β-CDs (HPCD) and hydroxypropyl-β-CDs coated with magnetic Fe_3_O_4_ nanoparticles (HPCD-EPI-MN), in combination with 100 µM methyl jasmonate (MJ), on **a** cell growth and **b** viability of transgenic *Vitis vinifera* stilbene synthase-expressing *Silybum marianum* cultures. **c** Cell viability assessed with fluorescein diacetate (**a**) and propidium iodide (**b**) in cultures treated with (1) 100 µM methyl jasmonate (MJ); (2) 100 µM methyl jasmonate in combination with 15 g/L of hydroxypropyl-β-CDs (HPCD); (3) 100 µM methyl jasmonate (MJ) in combination with 15 g/L hydroxypropyl-β-CDs coated with magnetic Fe_3_O_4_ nanoparticles (HPCD-EPI-MN) (3) for 120 h. Values in graphs are means ± SD of three independent replicates. For each treatment, values with different letters are significantly different (*p* < 0.05)

In the first 2 days after the addition of HPCD or HPCD-EPI-MN, no appreciable changes in cell growth were observed compared with the control. Prolonged incubation periods, however, affected both cell growth and viability, with cell death occurring in cultures treated with 15 g/L of HPCD-EPI-MN after a week. The effect of 100 µM MJ and different concentrations of HPCD or HPCD-EPI-MN in combination with 100 µM MJ on the cell growth and viability of *STS*-transformed *S.marianum* cultures is presented in Fig. 1.

**Fig. 2:**
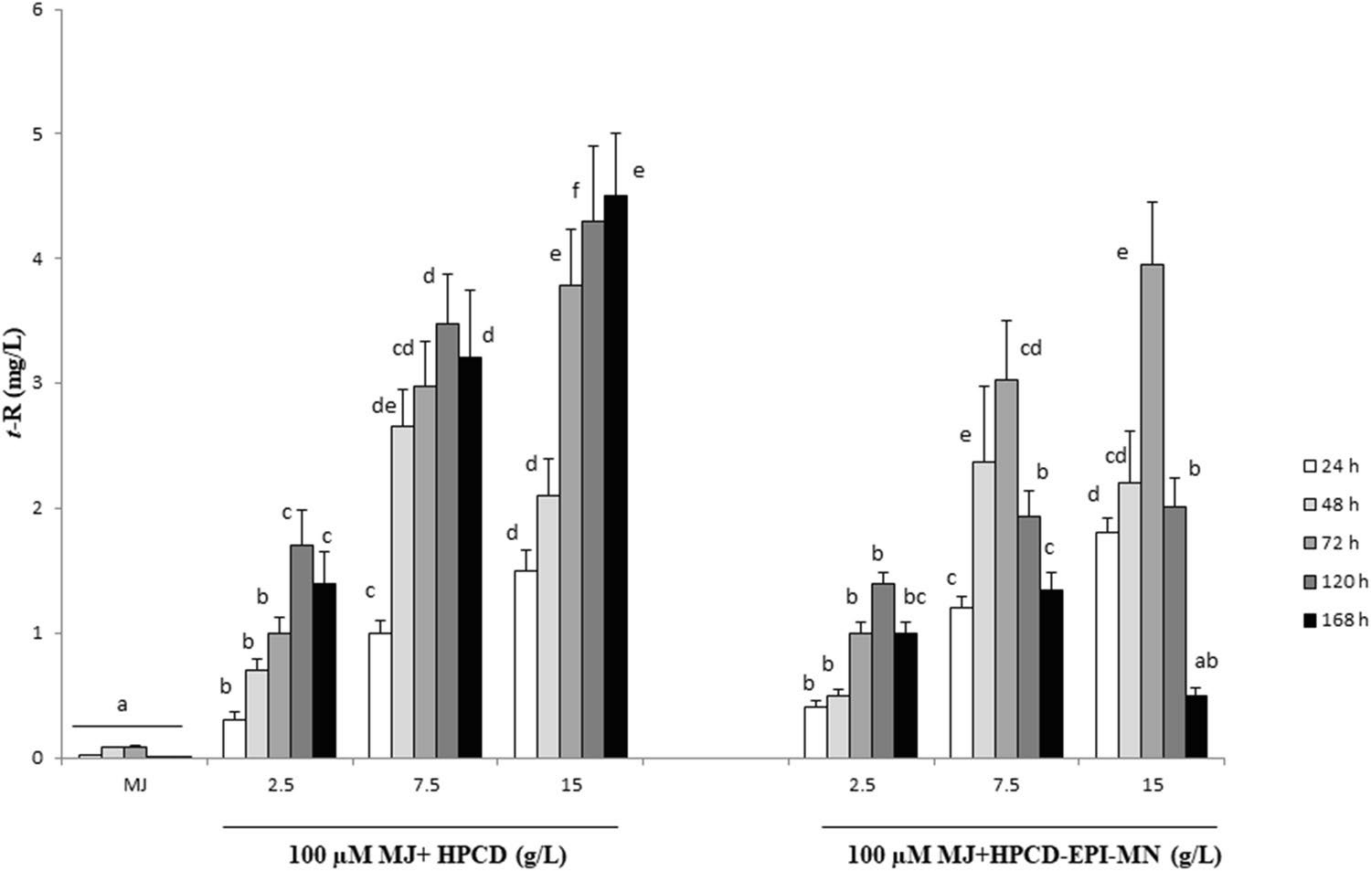
Extracellular accumulation of *t*-resveratrol in transgenic *Vitis vinifera* stilbene synthase-expressing *Silybum marianum* cultured cells treated with different concentrations of hydroxypropyl-β-CDs (HPCD) and hydroxypropyl-β-CDs coated with magnetic Fe_3_O_4_ nanoparticles (HPCD-EPI-MN), in combination with 100 µM methyl jasmonate (MJ) for different time periods. Values are means ± SD of three independent replicates. For each treatment, values with different letters are significantly different (*p* < 0.05)

As shown in Fig. 2, HPCD-EPI-MN promoted the accumulation of free *t*-R in the culture medium. Although levels were far lower than those reported in studies with other treatments, up to 4 mg/L of *t*-R was released in the presence of 15 g/L of HPCD-EPI-MN after 3 days. In the cultures treated with HPCD, similar amounts were detected, but production continued for longer, due probably to the preservation of the viability of cells under this treatment. On the other hand, in cultures treated only with MJ, very low levels of free *t*-R (an average of 50 µm/L) were found in the medium (see also Fig. 2). Figure S1 shows a chromatogram of extracts obtained from the culture medium of *STS*-transformed *S. marianum* cells treated with HPCD or HPCD-EPI-MN.

In contrast with the results reported in *V. vinifera*, in which HPCD-EPI-MN treatment was far more effective for *t*-R accumulation than the non-coated HPCDs (Almagro et al. 2020), the two types of CDs had a similar positive effect on *t*-R yields in the transgenic *S. marianum* cultures, possibly indicating a limited production capacity of the cell system.

**Fig. 3:**
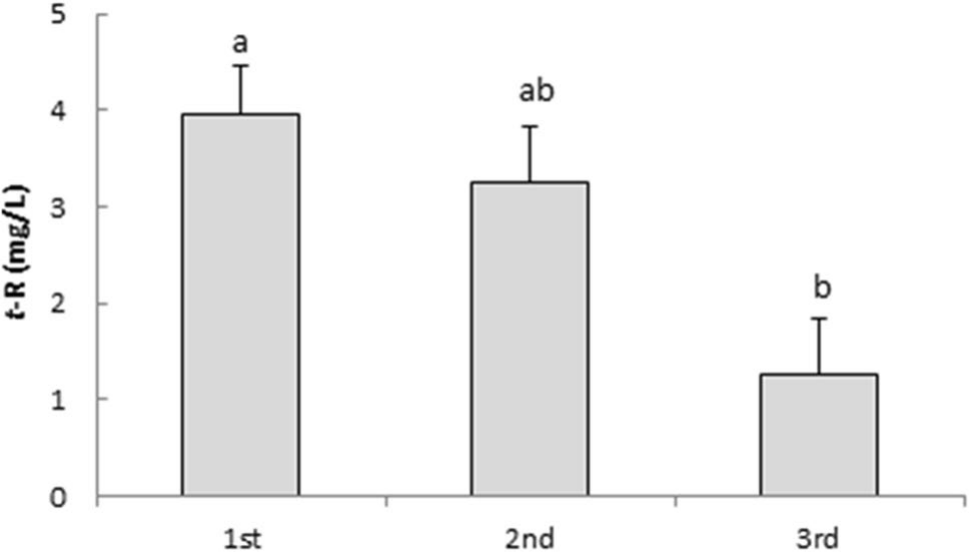
Variation in extracellular *t*-resveratrol production through three repeated elicitation cycles (72 h each) using the same recycled hydroxypropyl-β-CDs coated with magnetic Fe_3_O_4_ nanoparticles plus 100 µM methyl jasmonate. Values are means ± SD of three independent replicates. Values with different letters are significantly different (*p* < 0.05)

The recyclability of the HPCD-EPI-MN polymer was assessed in three consecutive elicitation cycles (72 h each), with the results shown in Fig. 3. HPCD-EPI-MN used twice preserved 80% of its initial efficiency, which indicates a good level of stability and reusability.

Cell growth and Ng production in *CHS*-transformed *S. marianum* cultures treated with HPCD or HPCD-EPI-MN.

**Fig. 4:**
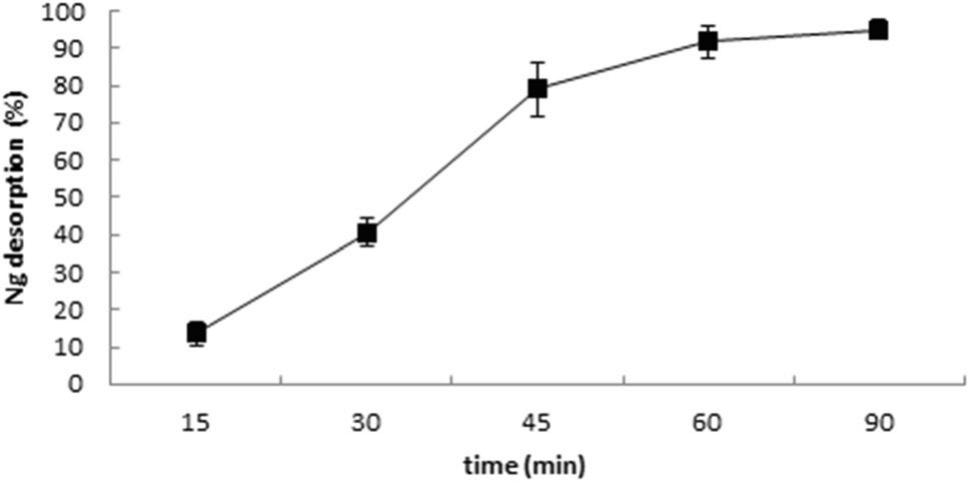
Ethyl acetate extraction time course of naringenin from hydroxypropyl-β-CDs coated with magnetic Fe_3_O_4_ nanoparticles. The desorption process was expressed as the percentage of naringenin recovered in ethyl acetate at each analyzed time analyzed. Bars: ± SD, *n* = 3

In previous in vitro studies, the use of HPCD increased Ng solubility, indicating that this complexation system is a viable option for its therapeutic oral delivery (Shulman et al. 2012). As for the case of *t*-R, in an in vivo preliminary analysis, extracellular Ng accumulation also occurred in cultures treated with MJ (100 µM) and 30 mM of HPCD. An average of 3.7 ± 0.28 mg/L of Ng was measured after 3 days, thus indicating the suitability of this class of modified CD for the experiments (Table S1). Our results show that HPCD-EPI-MN can also adsorb this commercially produced flavanone. After 30 min of stirring Ng with the magnetic polymer dispersed in water, as described in the “Material and methods” section, no residual Ng was detected in the aqueous solution. Moreover, almost complete Ng desorption from the polymer was achieved after 60 min of extraction with ethyl acetate (Fig. 4).

**Fig. 5:**
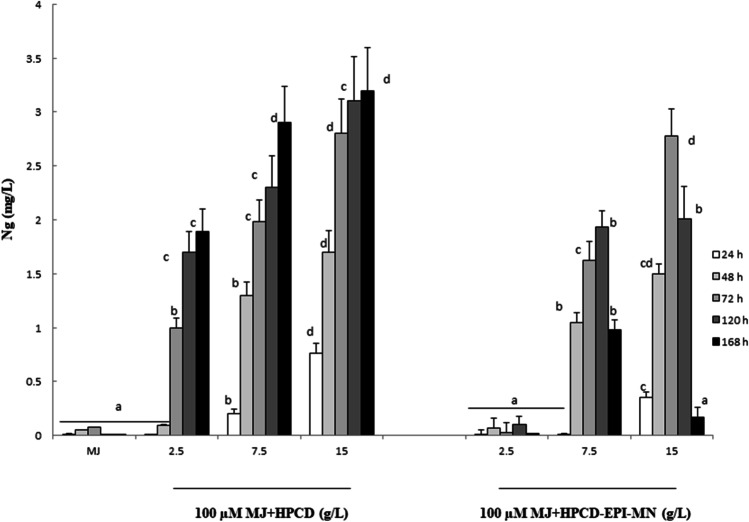
Extracellular accumulation of naringenin in transgenic *Cicer arietinum* chalcone synthase-expressing *Silybum marianum* cultured cells treated with different concentrations of hydroxypropyl-β-CDs (HPCD) and hydroxypropyl-β-CDs coated with magnetic Fe_3_O_4_ nanoparticles (HPCD-EPI-MN), in combination with 100 µM methyl jasmonate (MJ) for different time periods. Values in graphs are means ± SD of three independent replicates. For each treatment, values with different letters are significantly different (*p* < 0.05)

As shown in Fig. 5, treatment with both types of CDs induced the extracellular accumulation of free Ng in the *CHS*-transformed cultures; the highest values (approximately 3 mg/L) were obtained at day three in both HPCD- and HPCD-EPI-MN-treated cultures using the concentration of 15 g/L. Chromatograms of culture medium extracts from HPCD- or HPCD-EPI-MN-treated cultures are provided in Figure S2.

**Fig. 6:**
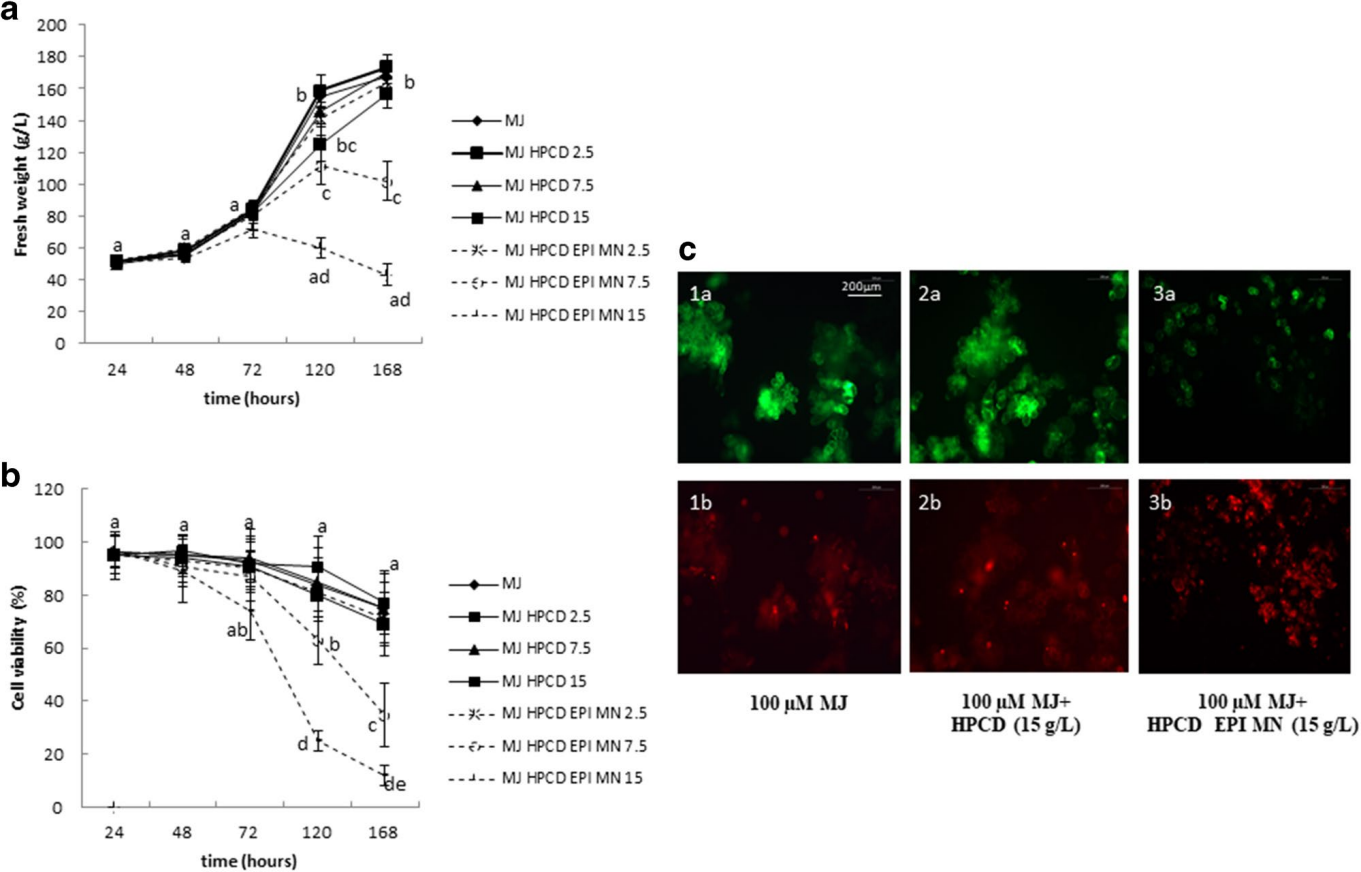
Effect of different concentrations (2.5, 7.5, and 15 g/L) of hydroxypropyl-β-CDs (HPCD) and hydroxypropyl-β-CDs coated with magnetic Fe_3_O_4_ nanoparticles (HPCD-EPI-MN), in combination with 100 µM methyl jasmonate (MJ), on **a** cell growth and **b** viability of transgenic *Cicer arietinum* chalcone synthase-expressing *Silybum marianum* cultures. **c** Cell viability assessed with fluorescein diacetate (**a**) and propidium iodide (**b**) in cultures treated with 100 µM methyl jasmonate (MJ) (1), 100 µM methyl jasmonate (MJ) in combination with 15 g/L of hydroxypropyl-β-CDs (HPCD) (2) and 100 µM methyl jasmonate (MJ) in combination with 15 g/L hydroxypropyl-β-CDs coated with magnetic Fe_3_O_4_ nanoparticles (HPCD-EPI-MN) (3) for 120 h. Values in graphs are means ± SD of three independent replicates. For each treatment, values with different letters are significantly different (*p* < 0.05)

The response of *CHS*-transformed cultures to HPCD-EPI-MN was similar to that of *STS*-transformed cultures, with growth and cell viability reduced in a concentration- and time-dependent manner. The highest tested dose (15 g/L) resulted in cell death after a week. Figure 6 shows the data for cell growth and viability of the cell suspension cultures under the different treatment regimes over 168 h.

**Fig. 7:**
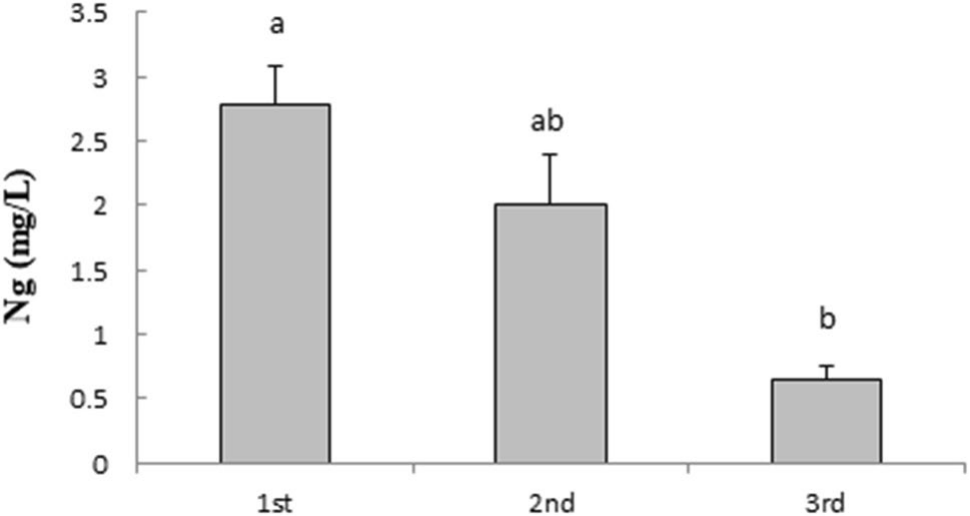
Variation in extracellular naringenin production through three repeated elicitation cycles (72 h each) using the same recycled hydroxypropyl-β-CDs coated with magnetic Fe_3_O_4_ nanoparticles plus 100 µM methyl jasmonate. Values are means ± SD of three independent replicates. For each treatment, values with different letters are significantly different (*p* < 0.05)

Similar to the results obtained for *t*-R, HPCD-EPI-MN reused once and twice retained 75 and 20% of its initial efficiency for stimulating Ng production, respectively (Fig. 7).

## Discussion

This study describes the positive influence of biosynthesized HPCD-EPI polymers coated with Fe_3_O_4_ nanoparticles on the production of *t*-R and Ng in transgenic *S. marianum* cell suspension cultures stably expressing an *STS* gene from *V. vinifera* and a *CHS* gene from *C. arietinum*, respectively. Additionally, the work reports for the first time the suitability of HPCDs, magnetized or not, to enhance the extraction of free Ng from cell cultures, thereby extending the data reviewed by Cardillo et al. (2021). The results confirm that CDs are a suitable strategy for improving bioprocesses in plant cell cultures, with extremely broad application.

As well as matching the ability of unmodified HPCDs to promote the extracellular accumulation of *t*-R and Ng, the inductive and adsorption capacity of HPCD-EPI-MN remained high for at least three elicitation cycles. These findings suggest that the magnetic polymers have promising application for reducing the costs of bioactive metabolite production in plant cell cultures. Only one study has previously experimented with CD-coated magnetic nanoparticles to boost *t*-R yields in plant cell suspensions, in that case, of *V. vinifera*. It was reported that after three cycles of elicitation, the magnetic polymers retained 45% of their original induction and adsorption properties (Almagro et al. 2020).

Although the effect of CDs coated with Fe_3_O_4_ nanoparticles on product extraction has still been scarcely investigated in living cell systems, several studies have used these CD polymers for the in vitro absorption of plant metabolites. Thus, Gong et al. (2014) and Lungoci et al. (2019) used HPCD and sulfobutylether CD polymers modified with magnetic particles to form inclusion complexes with rutin and protocatechuic acid, respectively. Li et al. (2016) also observed that CDs-functionalized magnetic reduced graphene oxide composites selectively adsorbed naphthalene-derived phytohormones from fresh tomatoes. These evidences demonstrate that magnetized CDs can act in both in vivo and in vitro systems by forming inclusion complexes with different plant metabolites.

The reusability of CD-coated magnetic nanoparticles has also been reported in in vitro studies. Liu et al. (2017) showed that magnetic porous CD polymers were able to remove organic pollutants at 86.35% even after seven cycles, whereas CDs capped with graphene-magnetite nanocomposites retained 80% of their adsorption capacity for bisphenol-A in water after six cycles of reuse (Ragavan and Rastogi 2019).

With respect to the the effects of classical CDs on cell growth in cell cultures of different plant species, contradictory results are reported in the literature. Thus, while Durante et al. (2011) observed that 50 mM of DMCD did not alter the growth of *Artemisia annua*, Perassolo et al. (2016) and Zhou et al. (2015) found that *Morinda citrifolia* and *Catharanthus roseus* grew more when treated with 20 mM of HPCD and 10 mM of DMCD, respectively. In our cultures, as in those of *Daucus carota* (Miras et al. 2016) and *V. vinifera* (Belchi-Navarro et al. 2012; Almagro et al. 2020), growth was reduced in the presence of CDs. In the case of the magnetic CDs, when applied at 15 mg/L, cell viability was preserved in *V. vinifera* cultures (Almagro 2020), but the same concentration had a very different impact on the *STS*- and *CHS*-transformed *S. marianum* cultures, resulting in extense cell death at day seven. This constitutes a serious handicap if cultures are to be maintained for prolonged periods in the presence of such agents.

The cause of HPCD-EPI-MN toxicity is likely to be highly complex and related to its physico-chemical properties. However, the main underlying mechanism is probably the triggering of an exacerbated production of reactive oxygen species, resulting in oxidative stress-induced cell death. This effect has been reported for several nanoparticles in plants, as reviewed by Rivero-Montejo et al. (2021).

In summary, although to date only limited literature is available on the effects of CD-coated nanoparticles on secondary metabolite pathways in plant cells, the present study shows that magnetic CD polymers can be used as elicitors to improve the biosynthesis and extraction of bioactive compounds. Moreover, their straightforward handling and recyclability indicates they have potential application as efficient tools for the commercial exploitation of plant cell cultures. More studies on the interaction between plant cells and CD-coated magnetic nanomaterials in terms of dosage, time of exposure and recyclability, among other factors, are necessary to improve and extend the applicability of this innovation.

Overall, the results of this study support that CD-coated magnetic nanoparticles represent an efficient and cheaper alternative to classical CD elicitors for the production of plant metabolites, in this case, two phenylpropanoid bioactive compounds, *t*-R and Ng. The addition of HPCD-EPI-MN (15 mg/L) proved to be an effective strategy for significantly increasing yield and excretion from the cells to the medium. The metabolite extraction and purification process are easy to carry out and HPCD-EPI-MN can be recycled, factors that could facilitate the establishment of a cell system for continuous production.

## Supplementary Information

Below is the link to the electronic supplementary material.

Supplementary file1 (PDF 205 KB)

## Data Availability

The authors confirm that the datasets supporting the findings and conclusions of this study are available within the article and its supplementary information file.
