# Supplementary material for: Phenylpropanoids in Silybum marianum cultures treated with cyclodextrins coated with magnetic nanoparticles
Source: Appl Microbiol Biotechnol. 2022 Mar 28;106(7):2393–401. doi: 10.1007/s00253-022-11886-2 (PMC8989811; doi:10.1007/s00253-022-11886-2)
Supplement: Supplementary file 1 — Supplementary file1 (PDF 205 KB) [file 253_2022_11886_MOESM1_ESM.pdf]

Phenylpropanoids in *Silybum marianum* cultures treated with cyclodextrins coated with magnetic nanoparticles

Purificación Corchete [1*], Lorena Almagro [2], María Angeles Pedreño [2], Javier Palazón [2]

[1]Laboratorio de Fisiología Vegetal. Facultad de Biología. Universidad de Salamanca, Spain

[2] Departamento de Fisiología vegetal. Facultad de Biología. Universidad de Murcia, Spain

[3] Laboratori de Fisiologia Vegetal, Facultat de Farmacia, Universitat de Barcelona, Spain

Corresponding author *

Purificación Corchete.

Email: corchpu@usal.es

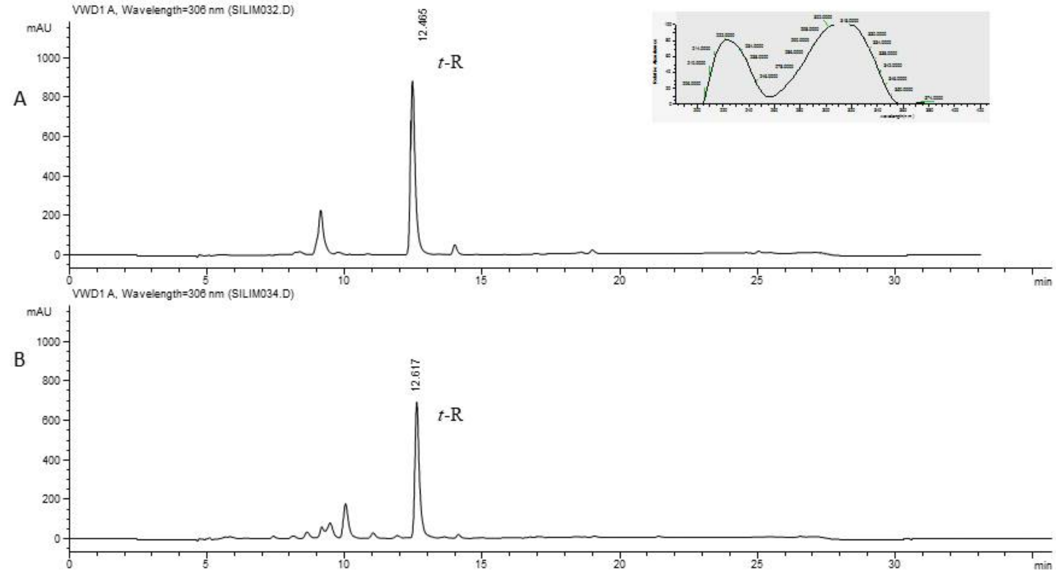

Supp **Fig.S1** Chromatogram (A306) of medium extracts of *Silybum marianum* cultures transformed with a *Vitis vinifera* stilbene synthase gene. A, cultures treated with 100 µM MJ and 15 g/L hydroxypropyl-β-CDs for 3 days . B, cultures treated with 100 µM MJ and 15 g/L hydroxypropyl-β-CDs coated with magnetic Fe_3O_4 nanoparticles for 3 days. Inset: UV spectra of the peak corresponding to *t*-resveratrol at retention time of 12.4 min.

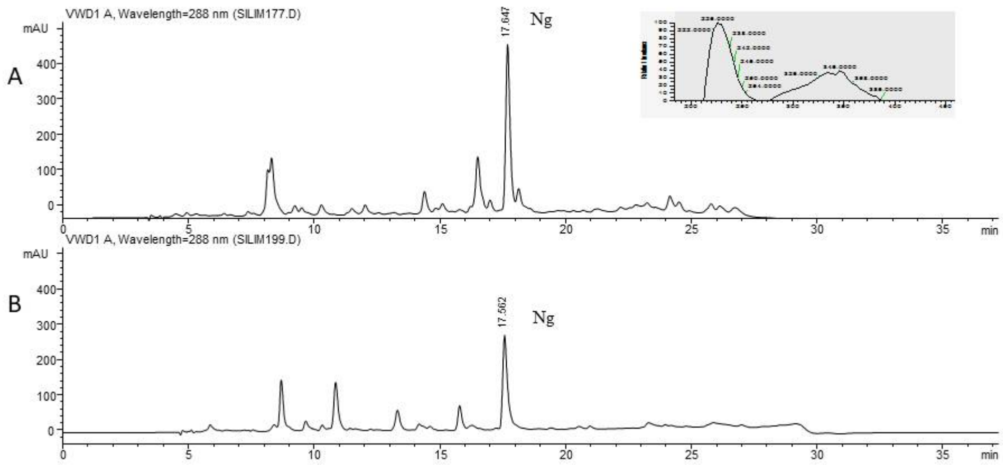

Supp **Fig. S2** Chromatogram (A288) of a medium extracts of *Silybum marianum* cultures transformed with *Cicer arietinum* chalcone synthase gene. A, Cultures treated with 100 µM MJ and 15 g/L hydroxypropyl-β-CDs for 3 days . B, Cultures treated with 100 µM MJ and 15 g/L hydroxypropyl-β-CDs coated with magnetic Fe_3O_4 nanoparticles for 3 days. Inset: UV spectra of the peak corresponding to naringenin at retention time of 17.5 min.

Supp **Table S1** Effect of hydroxypropyl-β-CDs (30mM) on extracellular accumulation of *t*-resveratrol and naringenin in transgenic *Silybum marianum* cultures elicited with 100 µM methyl jasmonate.

	t-resveratrol mg/L	naringenin (mg/L)
MJ	0.075± 0.008	0.015±0.001
MJ + HPCD	10.63 ± 1.8	3.7±0.28

Metabolites were analysed after three days of treatment. Results are means ±SD of three independent replicates.
